# Cortical responses elicited by luminance and compound stimuli modulated by pseudo-random sequences: comparison between normal trichromats and congenital red-green color blinds

**DOI:** 10.3389/fpsyg.2015.00053

**Published:** 2015-01-28

**Authors:** Bárbara B. O. Risuenho, Letícia Miquilini, Eliza Maria C. B. Lacerda, Luiz Carlos L. Silveira, Givago S. Souza

**Affiliations:** ^1^Instituto de Ciências Biológicas – Universidade Federal do ParáBelém, Brazil; ^2^Núcleo de Medicina Tropical – Universidade Federal do ParáBelém, Brazil; ^3^Universidade CeumaSão Luís, Brazil

**Keywords:** evoked potential, pseudo-random VECP, pattern-onset VECP, pattern-reversal VECP, color vision, trichromacy, daltonism

## Abstract

Conventional pattern-reversal visual evoked cortical potential (VECP) shows positivity for luminance and chromatic equiluminant stimuli while conventional pattern-onset VECP shows positivity for luminance pattern-onset and negativity for chromatic pattern-onset. We evaluated how the presentation mode affects VECPs elicited by luminance and compound (luminance plus chromatic) pseudo-random stimulation. Eleven normal trichromats and 17 red-green color-blinds were studied. Pattern-reversal and pattern-onset luminance and compound (luminance plus red-green) gratings were temporally modulated by m-sequence. We used a cross-correlation routine to extract the first order kernel (K1) and the first and second slices of the second order kernel (K2.1 and K2.2, respectively) from the VECP response. We integrated the amplitude of VECP components as a function of time in order to estimate its magnitude for each stimulus condition. We also used a normalized cross-correlation method in order to test the similarity of the VECP components. The VECP components varied with the presentation mode and the presence of red-green contrast in the stimuli. In trichromats, for compound conditions, pattern-onset K1, K2.1, and K2.2, and pattern-reversal K2.1 and K2.2 had negative-dominated waveforms at 100 ms. Small negativity or small positivity were observed in dichromats. Trichromats had larger VECP magnitude than color-blinds for compound pattern-onset K1 (with large variability across subjects), compound pattern-onset and pattern-reversal K2.1, and compound pattern-reversal K2.2. Trichromats and color-blinds had similar VECP amplitude for compound pattern-reversal K1 and compound pattern-onset K2.2, as well as for all luminance conditions. The cross-correlation analysis showed high similarity between waveforms of compound pattern-onset K2.1 and pattern-reversal K2.2 as well as pattern-reversal K2.1 and K2.2. We suggest that compound pattern-reversal K2.1 is an appropriate response to study red-green color-opponent activity.

## INTRODUCTION

Using pseudo-random stimulation it was possible to elicit negative-dominated visual evoked cortical potentials (VECP) for chromatic equiluminant contrast using both pattern-onset and pattern-reversal stimuli (Gerth et al., 2003). These findings differed from those obtained with conventional periodical stimulation (Carden et al., 1985; Suttle and Harding, 1999). When conventional periodical stimuli are used, such as sinusoidal gratings, luminance, and chromatic equiluminant pattern-reversal as well as luminance pattern-onset stimulation elicited positive-dominated VECP at around 100 ms while chromatic equiluminant pattern-onset stimulation elicited negative-dominated VECP at the same latency (Carden et al., 1985; Kulikowski and Parry, 1987; Murray et al., 1987; Kulikowski et al., 1989). Since the chromatic equiluminant pattern-onset VECP usually shows higher signal-to-noise ratio compared to chromatic equiluminant pattern-reversal VECP and also exhibit inverse polarity compared to luminance pattern-onset VECP, many have used this stimulation mode to study the mechanism of chromatic equiluminant transient VECPs (Carden et al., 1985; Kulikowski and Parry, 1987; Murray et al., 1987; Berninger et al., 1989; Kulikowski et al., 1989, 1996; Rabin et al., 1994; Porciatti and Sartucci, 1999; Gomes et al., 2006, 2008, 2010; Souza et al., 2008).

McKeefry et al. (1996) discussed about the activation of chromatic and achromatic mechanisms by pattern reversal and pattern onset–offset stimulations. They based their suggestions in the features of response from tonic and phasic cells in the visual system (Gouras, 1968; Dreher et al., 1976; Kaplan and Shapley, 1982, 1986; Hicks et al., 1983). They consider that for some stimulus selective condition the dichotomy between tonic/sustained cells and phasic/transient cells overlap with magnocellular (luminance) and parvocellular (chromatic) activity, respectively. Tonic cells has larger responses for the onset of the stimulus than for its offset due the sustained response is longer as long as the stimulus is on, and it has low responsivity to contrast reversal modulated by temporal square-wave function due its sustained nature of their response. Phasic cells had similar responses for the stimulus onset and offset due their transient response, but they has higher responses for contrast reversal with square-wave temporal modulation. McKeefry et al. (1996) found that the chromatic onset–offset VECP is dominated by a fundamental component, while the achromatic onset–offset VECP had a second harmonic additionally to the fundamental component. For pattern reversal VECPs, both chromatic and achromatic responses, had high second harmonic, which peaked at the achromatic stimulus condition and was minimum at the chromatic isoluminance.

Until now, only homogenous fields or complex patterns were used to compose pseudo-random stimuli to investigate chromatic cortical responses in different visual field sectors (Baseler and Sutter, 1997; Gerth et al., 2003). For spatial vision, additionally to dartboard stimulus (Baseler and Sutter, 1997), a stimulus composed by a matrix of triangles was also used to elicit cortical activity in multifocal VECP studies (Gerth et al., 2003). Triangle patterns were used to reduce the high spatial frequency components present in other forms of stimulation. However, it is difficult to make a straightforward association between the spatial properties of triangle patterns or dartboard patterns and those of sinusoidal gratings that are relevant for the recorded cortical responses. Sinusoidal gratings are the simplest stimuli used to study spatial vision and they were widely used in intracellular and extracellular single-unit electrophysiology, non-invasive electrophysiology, and psychophysics (e.g., Carden et al., 1985; Mullen, 1985; Lee et al., 1989).

Gerth et al. (2003) showed that chromatic pseudo-random VECP had the same polarity nevertheless the presentation mode used to elicit the cortical response, but in their study it lacking the comparison with luminance VECPs. Based in the findings of the effects of the pattern mode presentation on conventional VECPs we could expect that different pseudo random VECP waveforms would be elicited by achromatic and chromatic stimulus for trichromat observers, and color-blind subjects would have decrease or absent responses for chromatic stimulation.

Some studies indicated that the use of non-linear analysis with the separation of the visual response in different states of adaptation (kernels) permit to investigate the presence of mechanisms in the cortical response with different physiological properties distributed in the different kernels (Crewther and Crewther, 2010; Araújo et al., 2013). In the present study, we applied the paradigm of Gerth et al. (2003) by using sinusoidal gratings in order to evaluate how the presentation mode affects luminance and chromatic pseudo-random VECPs. Responses obtained from normal trichromats and red-green congenital color-blinds were compared. A short communication comprising some results of this work was previously presented in the ARVO Annual Meeting (Souza et al., 2012).

## MATERIALS AND METHODS

### SUBJECTS

All procedures were approved by the Ethic Research Committee of the Tropical Medicine Nucleus, Federal University of Para (Protocol #023/2011). Eleven normal trichromats (21.28 ± 1.86 years old) and 17 red-green congenital color-blinds (eight protans and nine deutans) were monocularly tested. None of the subjects had previous visual or neurological diseases. We evaluated subject color vision using the Ishihara Plates (1997 38-plate edition; Kanehara & Co Ltd, Tokyo, Japan), an anomaloscope (HMC-Anomaloskop model 47715, Oculus Optikgeräte GmbH, Wetzlar, Germany), and by measuring their color discrimination thresholds (Cambridge Colour Test, Cambridge Research System Ltd, Rochester, England, UK).

### STIMULATION

Visual stimulation, bioelectric recording, and data extraction were performed using a Veris Science 6.10 system (Electro-Diagnostic Imaging, Inc., Redwood City, CA, USA). We presented luminance and compound horizontal gratings, 8°of visual angle, 2 cycles/degree in a CRT display with 75 Hz frame rate and 1280 × 1204 pixels spatial resolution (FlexScan T662, Eizo, Ishikawa, Japan).

We used sine-wave gratings for the luminance test. A yellow chromaticity (CIE 1976 color space: u’ = 0.276, v’ = 0.545) was modulated with 99% Michelson contrast, mean luminance of 10 cd/m^2^. The background was a homogeneous field with the same chromaticity and mean luminance.

**FIGURE 1:**
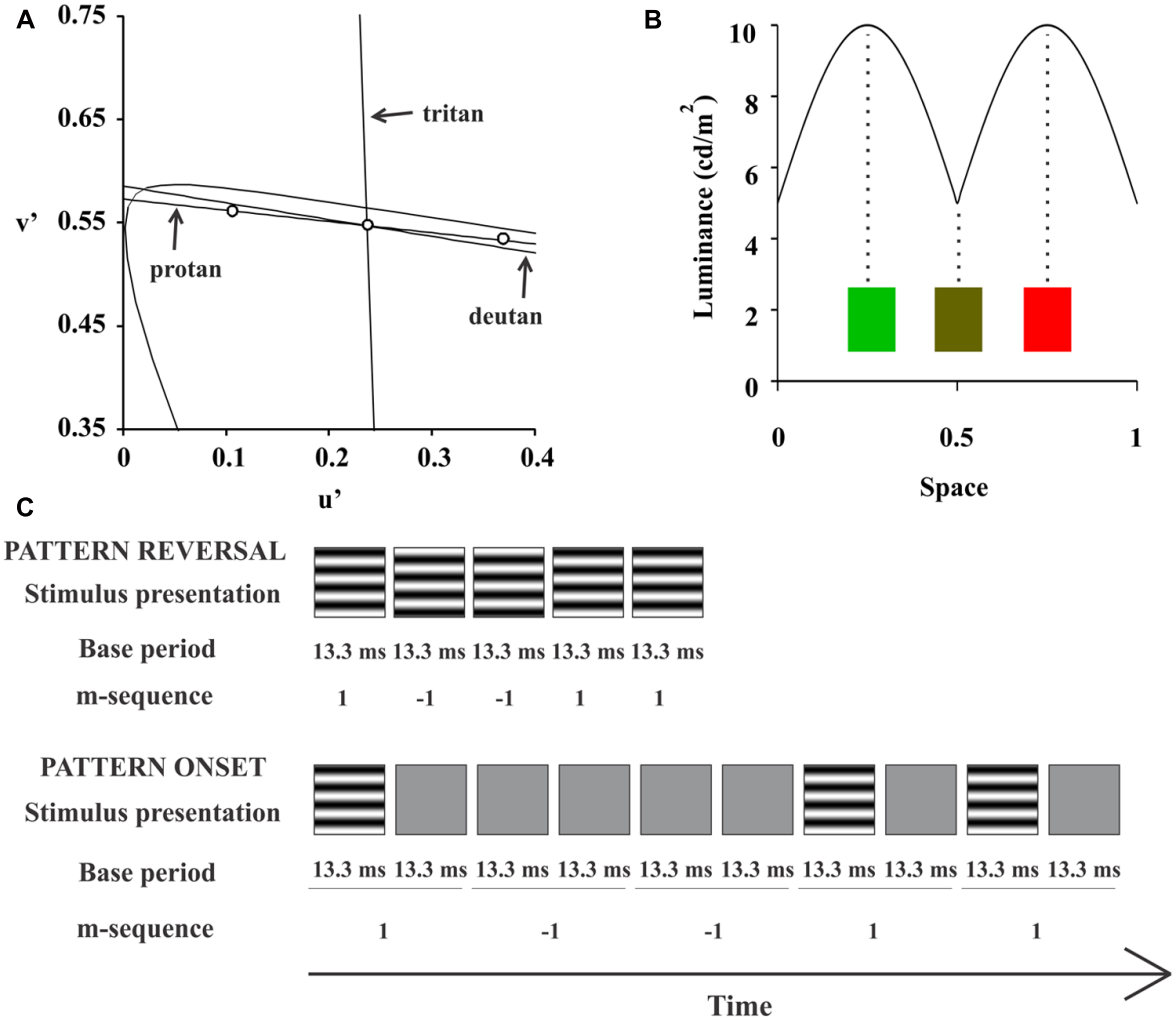
**Compound stimuli.** For compound stimulation, we used two chromaticities defined in the CIE 1976 Chromaticity Diagram: red (u’ = 0.432, v’ = 0.527) and green (u’ = 0.12, v’ = 0.564; **(A)** For each half cycle of the stimulus, the luminance changed sinusoidally from 5 to 10 cd/m^2^ and back, at the same time that the chromaticity changed sinusoidally from green or red to the intermediate yellow, **(B). (C)** Shows a space-time diagram of the m-sequence configuration for each presentation mode.

We used two chromaticities for the test with compound gratings, red (u’ = 0.432, v’ = 0.527) and green (u’ = 0.12, v’ = 0.564; **Figure 1A**). For each half cycle of the stimulus the luminance changed sinusoidally from 5 to 10 cd/m^2^ and back at the same time that the chromaticity changed sinusoidally from green or red to the intermediate yellow (**Figure 1B**). The chromatic contrast was modulates along a protan confusion line and at about five away from a deutan confusion line in the CIE 1976 color space. The background had the same stimulus yellow mean chromaticity but the luminance was kept at 10 cd/m^2^ throughout the entire stimulus set. For monitor calibration, we used a CS-100A Colorimeter (Minolta, Osaka, Japan). Compound gratings used in this study was similar but not entirely equal to those used previously by Lee et al. (2011) and Li et al. (2014).

A binary m-sequence (2^14^-1 elements) controlled stimulus temporal presentation. We used two stimulus presentation modes: pattern-reversal and pattern-onset. For pattern-reversal, each one of two m-steps had base period of 13.3 ms and we set each of them to show each frame of the grating stimulus with 180°phase difference. Most of the multifocal VECP studies used this configuration for pattern-reversal mode (e.g., Baseler and Sutter, 1997). For pattern-onset, each m-step had a base period of 26.6 ms (two frames). The first m-step presented one frame with the grating followed by another frame with the background. The second m-step showed two consecutive frames with the background. This pattern-onset configuration was used before to improve VECP signal-to-noise ratio (Hoffmann et al., 2003). Therefore, we had four stimulus conditions: luminance pattern-reversal, luminance pattern-onset, red-green pattern-reversal, and red-green pattern-onset. A space-time diagram of each pattern mode stimulation is shown in the **Figure 1C**.

### RECORDING SETTINGS

One-channel electroencephalographic signals were recorded using 10 mm gold surface electrodes (Grass Safelead Gold Disc Electrodes, Grass Technologies, Richmond, USA). Electrode placement followed the standard of the International Society for Clinical Electrophysiology of Vision (ISCEV; Odom et al., 2010): active electrode was placed at Oz, reference electrode at Fz, and ground electrode at Fpz. Continuous recordings were amplified x50,000, on-line filtered between 0.1 and 100 Hz (P511 Amplifier, Grass Technologies), and digitized at 1.2 kHz. For each stimulus condition, we used the Veris Science version 6.010 platform to perform a cross-correlation. The kernel extraction from the VECP was set using the Veris Science software. We extracted the first order kernel (K1), second order kernel first slice (K2.1), and second order kernel second slice (K2.2). The kernel for more information about kernel significance, see Sutter (2000) and Odom (2006). After kernel extraction, the waveforms were low-pass filtered at 50 Hz.

### DATA ANALYSIS

**FIGURE 2:**
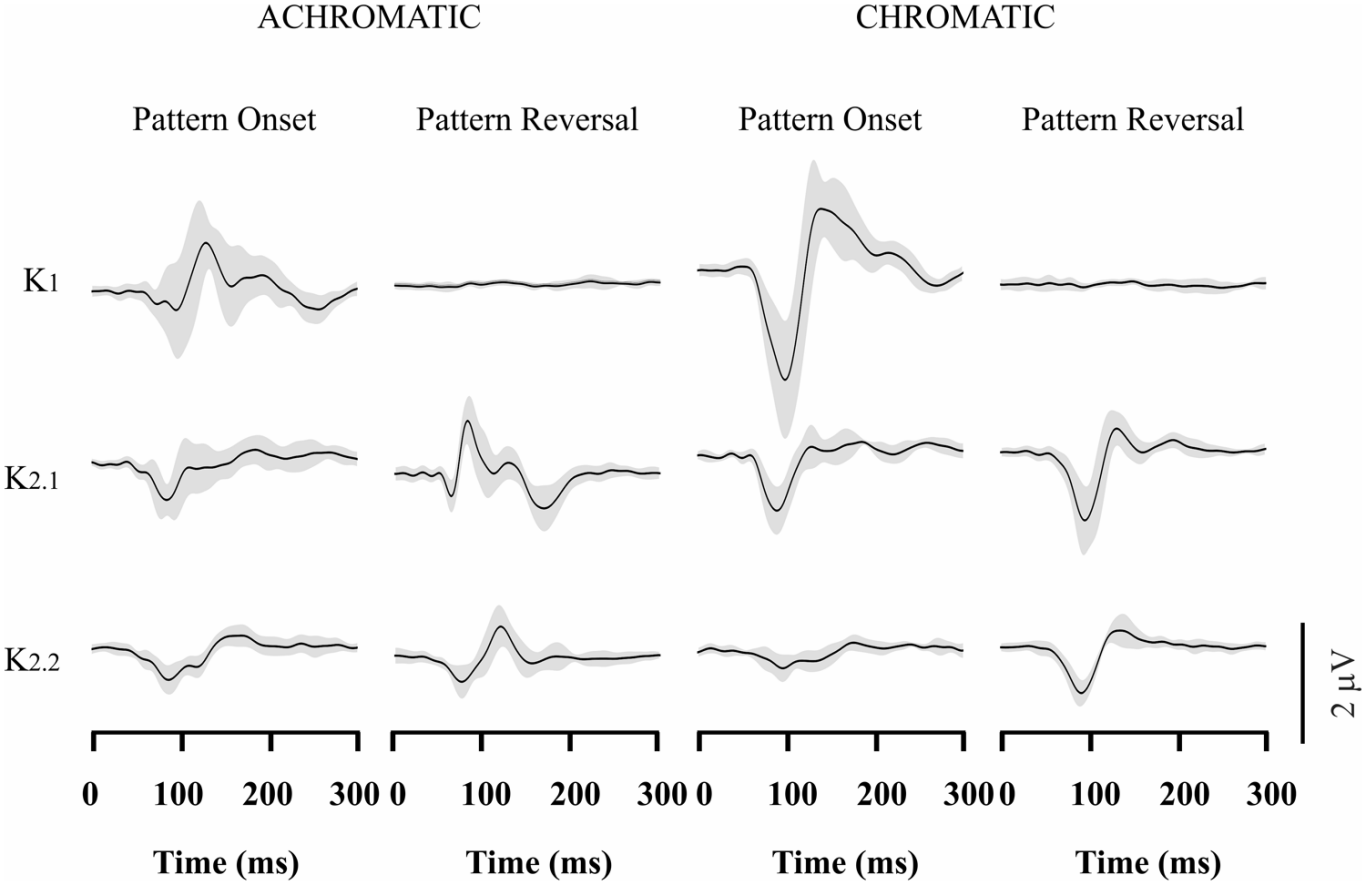
**Visual evoked cortical potential (VECP) average waveforms obtained from normal trichromats using luminance and compound stimuli, as well as pattern-onset and pattern-reversal stimulations.** Waveforms were obtained by averaging results from 11 subjects. From top to bottom are depicted waveforms corresponding to the first order kernel (K1), first slice of the second order kernel (K2.1), and second slice of the second order kernel (K2.2). The average waveforms were dominated by the presence of negativity at about 100 ms for the majority of stimulus conditions and kernels. An important exception is achromatic pattern-reversal K2.1 waveform which is dominated by a positive peak at the same latency. The gray shades represent the SD of the averaged recordings. They provide an indication of interindividual variability.

**FIGURE 3:**
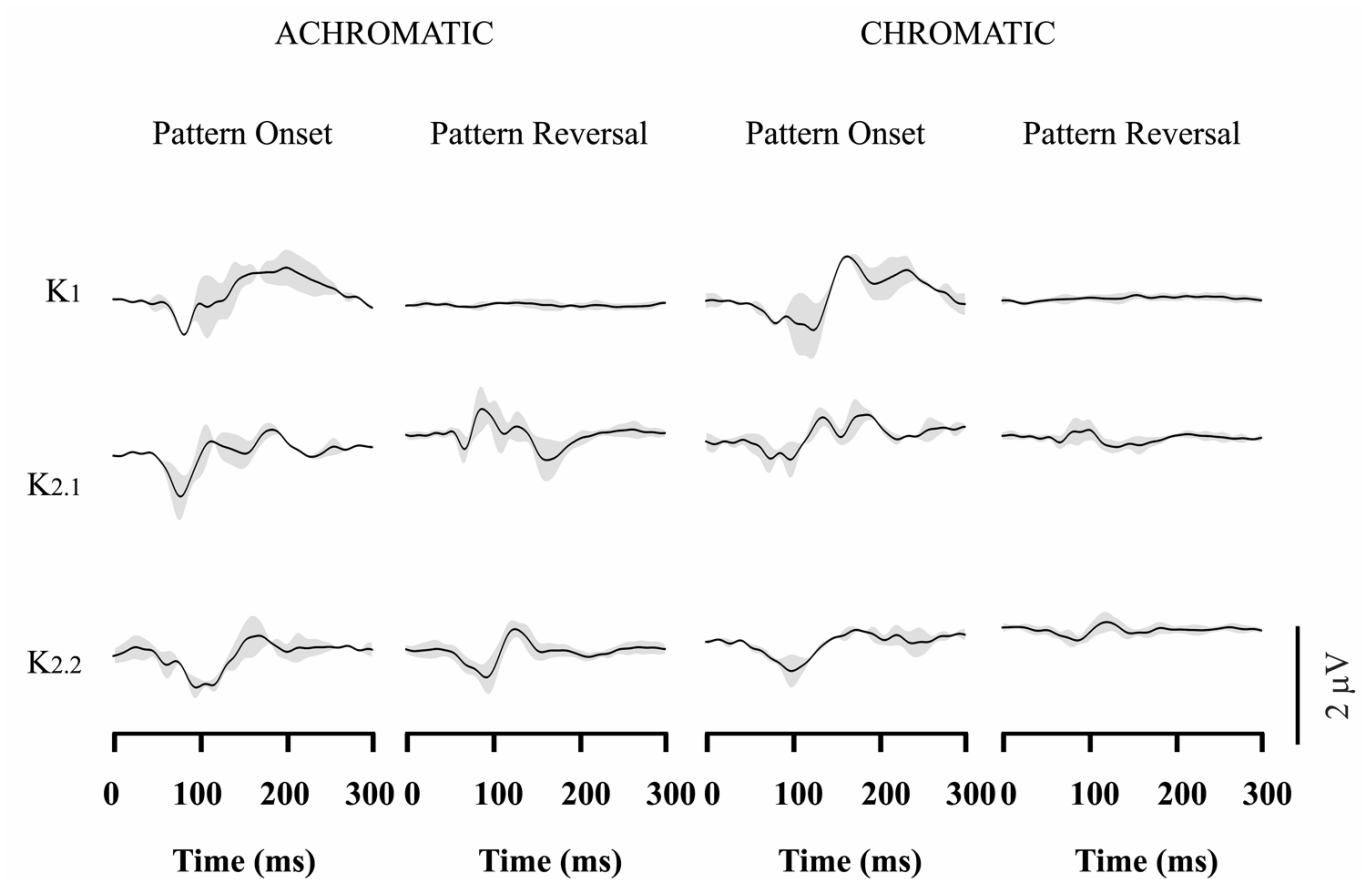
**Visual evoked cortical potential average waveforms obtained from protans using luminance and compound stimuli, as well as pattern-onset and pattern-reversal stimulations.** Waveforms were obtained by averaging results from eight subjects. For luminance stimuli, VECP waveforms were similar to those obtained from trichromats. For compound stimuli, pattern-onset stimulation elicited smaller negative amplitudes than in trichromats at 100 ms, while pattern-reversal stimulation evoked no response or small positive components at the same latency. The gray shades represent the SD of the averaged recordings. They provide an indication of interindividual variability.

**FIGURE 4:**
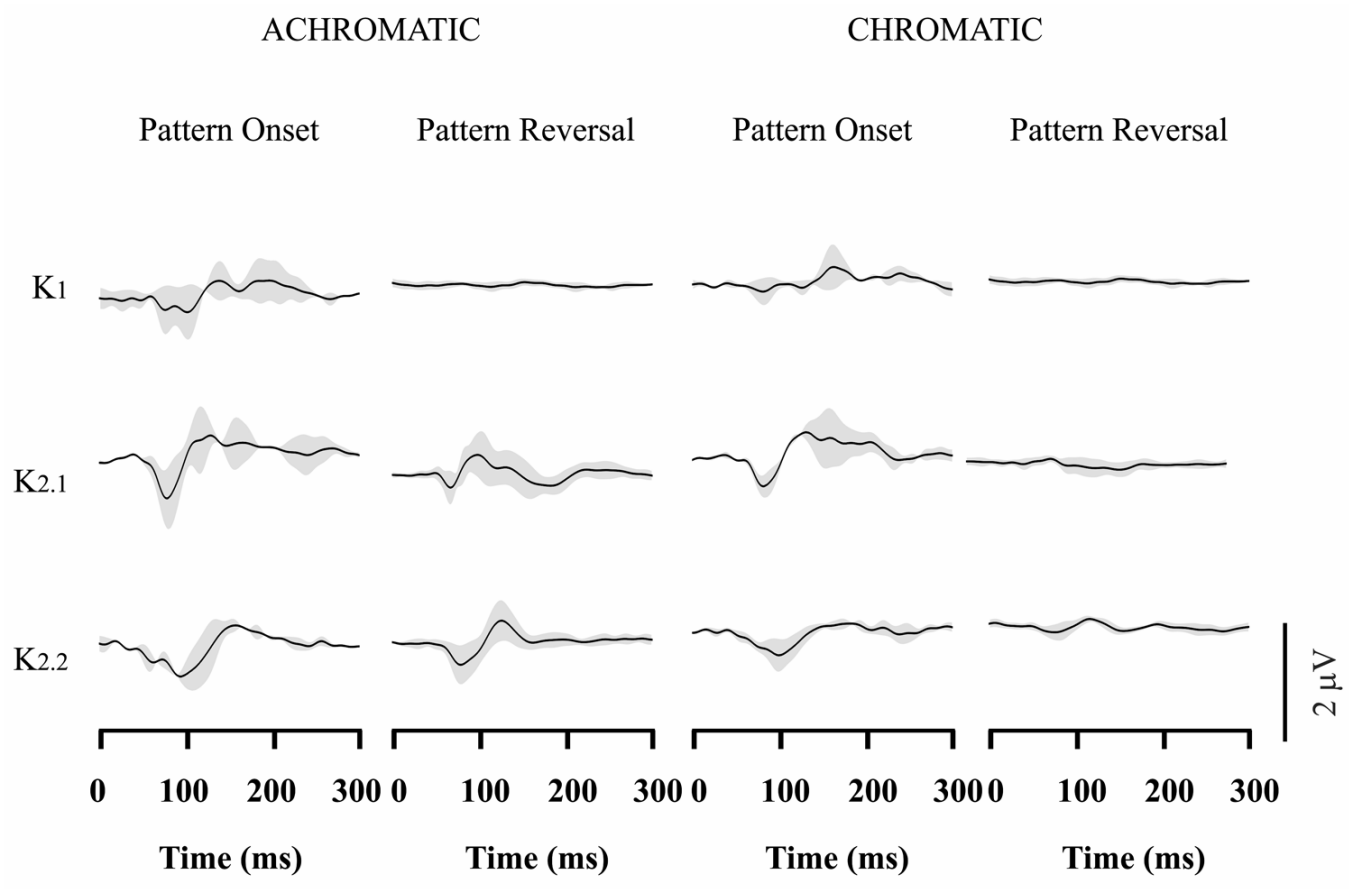
**Visual evoked cortical potential average waveforms obtained from deutans using luminance and compound stimuli, as well as pattern-onset and pattern-reversal stimulations.** Waveforms were obtained by averaging results from 9 subjects. For luminance stimuli, VECP waveforms were similar to those obtained from trichromats. For compound stimuli, pattern-onset stimulation elicited smaller negative amplitudes at 100 ms than in trichromats, while pattern-reversal stimulation resulted in no response or a small positivity at the same latency. The gray shades represent the SD of the averaged recordings. They provide an indication of interindividual variability.

Visual evoked cortical potential kernel waveforms had several positive and negative components starting at about 70 ms after stimulus onset (**Figures 2–4**). In order to estimate the evoked response magnitude, we calculated the recording total power. The total power was taken as the numerical integration of the squared amplitude of all amplitude data in the first 500 ms of the recordings. We used Kruskal–Wallis test with Dunn’s *post hoc* test (α = 0.05) to compare the magnitude of the VECP components at different stimulus conditions. The *p*-values were corrected by Bonferroni correction.

## RESULTS

### VECP WAVEFORMS IN TRICHROMATS

The VECP waveforms across different kernels varied with stimulus presentation mode and presence of color contrast in the stimulus. **Figure 2** shows the VECP kernels waveforms elicited by different stimuli in trichromats. Results obtained from the 11 trichromats were averaged to provide **Figure 2** waveforms. For luminance pattern-onset stimulation, K1 had a negative peak at about 100 ms followed by a positive peak at about 150 ms. K2.1 and K2.2 also had a negative peak at 100 ms, but the positivity was small or entirely missing. For luminance pattern-reversal stimulation, K1 was very small or absent, K2.1 had a negative peak at about 85 ms followed by a double-peaked positivity between 100 and 120 ms, and K2.2 had a negative peak at 100 ms followed by a positive peak at 130 ms.

For compound pattern-onset stimulation, all kernels waveforms (K1, K2.1, and K2.2) were dominated by negativity at about 100 ms. K1 also had a pronounced positive peak at 150 ms following the very pronounced main negative component. For compound pattern-reversal, similarly to luminance pattern-reversal stimulation stimulation, K1 was also very small or absent. In addition, K2.1 and K2.2 were dominated by a negativity occurring at about 100 ms. It should be noted that K2.1 showed opposite polarity for compound pattern-reversal stimulation when compared with luminance pattern-reversal stimulation. It can be interpreted as an indication of differential activation on luminance and chromatic mechanism.

### VECP WAVEFORMS IN CONGENITAL RED-GREEN COLOR BLINDS

**Figures 3–4** show VECP waveforms for different kernels obtained by recording from red-green congenital color blinds, either protans (**Figure 3**) or deutans (**Figure 4**). As for normal trichromats (**Figure 2**), VECP was elicited by luminance and compound stimuli as well as by pattern-onset and pattern-reversal stimulation modes. Waveforms were obtained by averaging the results obtained from eight protans and nine deutans, respectively.

Red-green color blinds responses were similar to responses obtained from normal trichromats for both kinds of luminance stimulation. For luminance pattern-onset stimulation, K1 had low-amplitude negative and positive components (slightly larger in protans when compared with deutans), while K2.1 and K2.2 were dominated by a negative peak at about 100 ms. For luminance pattern-reversal stimulation, all kernels were very similar in color blinds and normal trichromats: K1 was very small or absent, K2.1 had a negative peak at about 85 ms followed by a double-peaked positivity between 100 and 120 ms, and K2.2 had a negative peak at 100 ms followed by a positive peak at 130 ms.

For compound pattern-onset, K1 was very small or had a small negativity at about 100 ms in different color-blind subjects, was much smaller in both groups of color blinds when compared with normal trichromats, and was larger in protans when compared with deutans. K2.1 and K2.2 were dominated by a negative peak at 100 ms. K2.1 was small in deutans, slightly larger in protans, and larger in normal trichromats. K2.2 was similar in the three groups.

For compound pattern-reversal, K1 was absent while K2.1 and K2.2 were very small with a small positive component at 100 ms in both groups of color blinds. Thus, while pattern-reversal K1 was similar in normal trichromats, protans, and deutans, pattern-reversal K2.1 and K2.2 were very different between color blinds and normal trichromats.

### EVALUATION OF THE VECP MAGNITUDE AND ITS COMPARISON AMONG THE KERNELS

**FIGURE 5:**
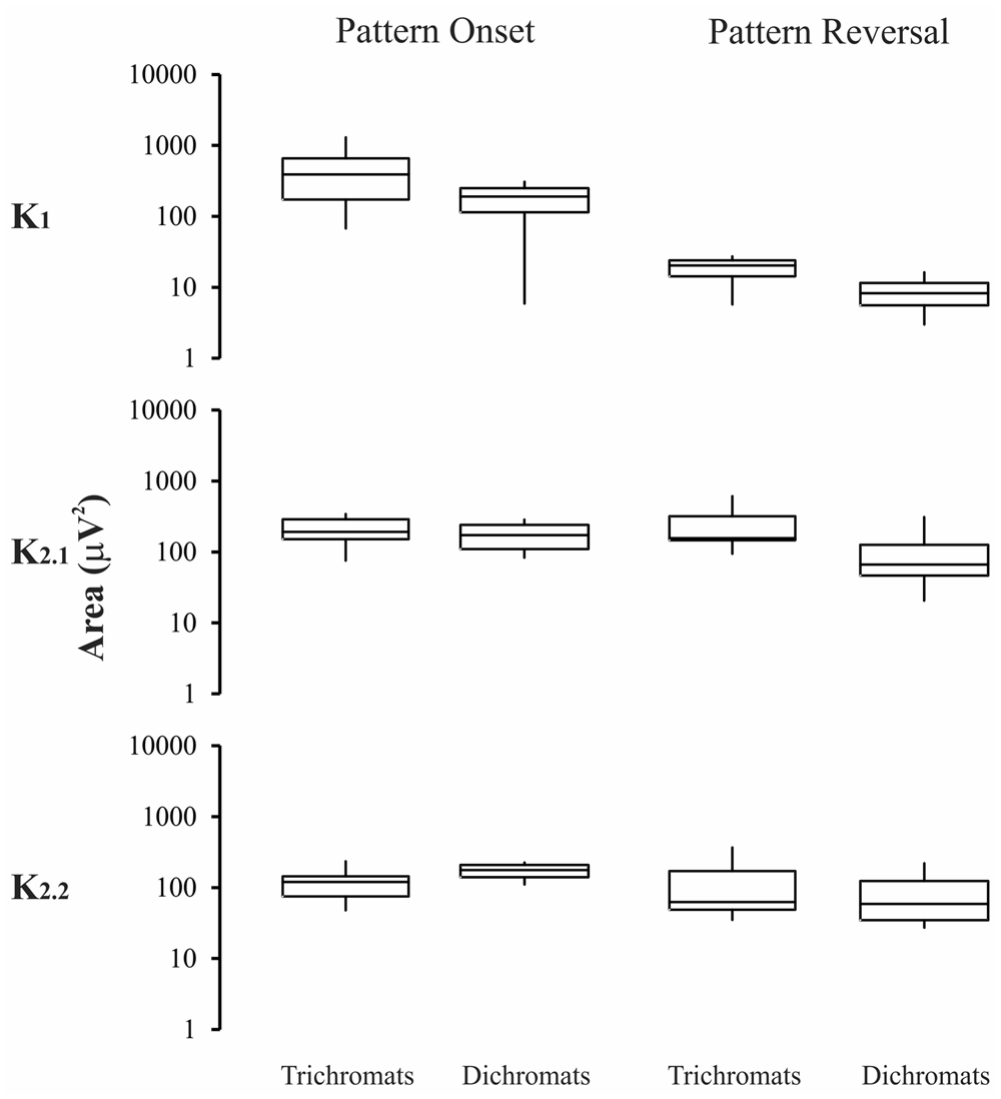
**Magnitude of the VECP elicited by luminance stimuli in trichromats and color blinds.** No difference was found in the results obtained with pattern-onset and pattern-reversal for all kernels waveforms. Box-plots represent medians (middle horizontal lines), first quartiles (lower horizontal lines), third quartiles (upper horizontal lines), maximum values (upper vertical lines), and minimum values (lower vertical lines).

**FIGURE 6:**
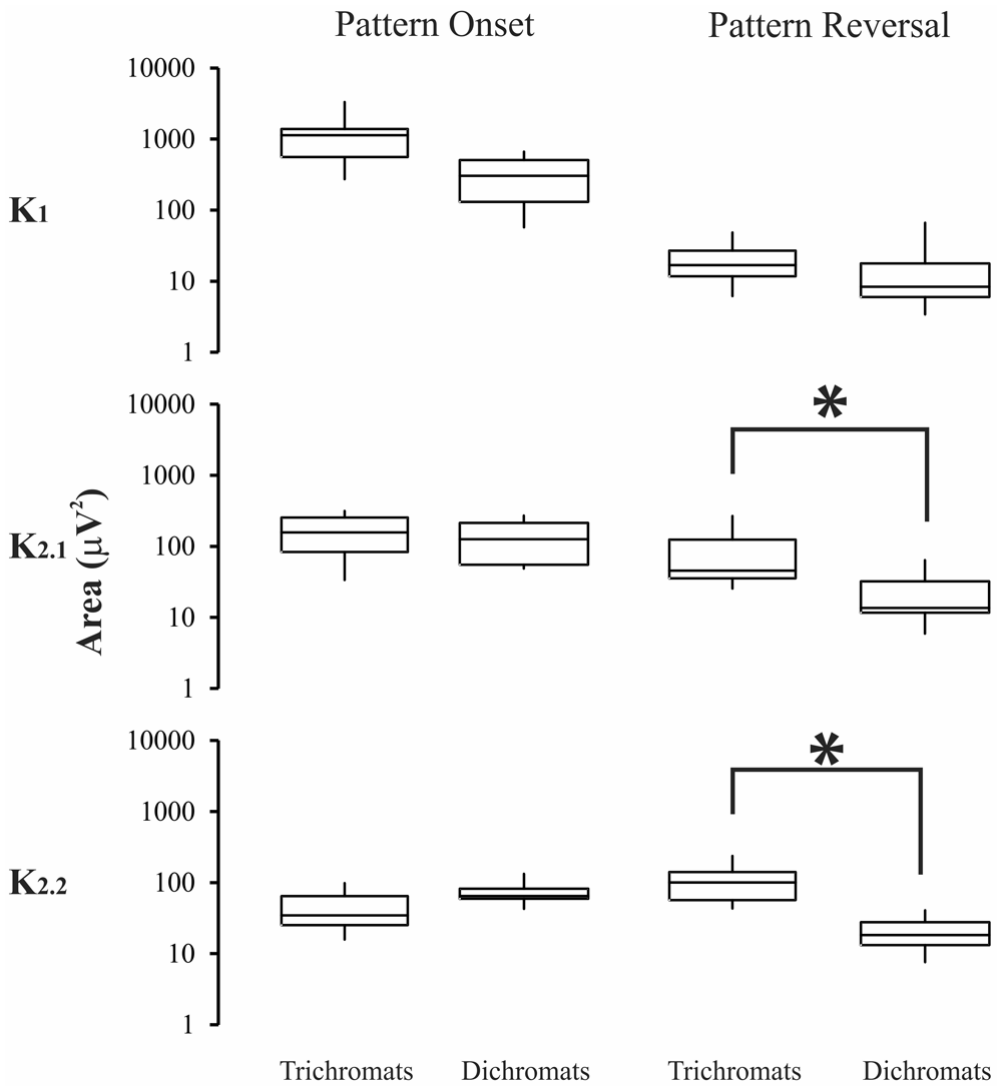
**Magnitude of the VECP elicited by compound stimuli in trichromats and color blinds.** Pattern-reversal K2.1, and pattern-reversal K2.2 had higher values for trichromats than color-blinds. Asterisks (*) were used to mark statistically significant differences. Box-plots represent medians (middle horizontal lines), first quartiles (lower horizontal lines), third quartiles (upper horizontal lines), maximum values (upper vertical lines), and minimum values (lower vertical lines).

**Figures 5–6** show box-plots representing the recording total power in the first 500 ms elicited by luminance (**Figure 5**) or compound (**Figure 6**) stimuli. Results from normal trichromats are compared with those from red-green color-blinds dichromats. Protans and deutans were grouped together for this comparison with normal trichromats. For luminance stimuli (**Figure 5**) there were no significant differences (*p* > 0.05) for both pattern-onset and pattern-reversal stimulation modes and for the three kernels: pattern-onset K1, *p* = 0.7; pattern-onset K2.1, *p* = 0.16; pattern-onset K2.2, *p* = 0.35; pattern-reversal K1, *p* = 0.47; pattern-reversal K2.1, *p* = 0.12; pattern-reversal K2.2, *p* = 0.98.

For compound stimuli (**Figure 6**) trichromats had significant larger VECP magnitude than color-blinds in the pattern-reversal K2.1, and pattern-reversal K2.2 (*p* < 0.05). The results of the statistical analysis were as follows: pattern-onset K1, *p* = 0.09; pattern-onset K2.1, *p* = 0.12; pattern-onset K2.2, *p* = 0.64; pattern-reversal K1, *p* = 0.57; pattern-reversal K2.1, *p* = 0.002; pattern-reversal K2.2, *p* = 0.0001).

For the dichromats, the kernels of the chromatic waveforms were very similar to those of the achromatic waveforms with slightly reduced amplitude. However, there were no significant differences in amplitudes between the same kernel (or kernel slice).

## DISCUSSION

We used pseudo-random sequences to temporally modulate luminance and compound (luminance plus red-green) sinusoidal gratings presented in pattern-onset and pattern-reversal modes. The present study introduced the use of compound gratings to study the VECP mechanisms. Previously, similar stimuli were used in psychophysical, electroretinographic, and single-unit studies (Lee et al., 2011; Parry et al., 2012; Li et al., 2014). The VECP kernels elicited by pattern-onset and pattern-reversal compound gratings was dominated by negative components at 100 ms. This kind of compound stimuli was previously used in order to simultaneously generate visual responses for luminance and chromatic contrast (Lee et al., 2011). In the present study, we suggest that the responses for compound stimuli were dominated by chromatic information, especially for pattern-reversal stimulation. Two results support this hypothesis: (i) the pattern-reversal luminance response was dominated by a positive component, while the pattern-reversal compound response was dominated by negativity at the same latency; and (ii) compound stimuli elicited small or no response in red-green congenital color blinds indicating that the cortical response for compound stimuli in the present experiment was dominated by chromatic contribution. Even compound stimuli have been composed by luminance plus chromatic contrast the responses for the present experiments seem to be dominated by chromatic information.

Carden et al. (1985), using conventional VECP, described luminance and chromatic pattern-reversal VECP dominated by positivity at 100 ms and pattern-onset VECP dominated by negativity for chromatic stimuli and positivity for luminance stimuli at the same latency. We have observed that only pattern-reversal stimuli elicited K2.1 with opposite polarity at 100 ms for luminance or compound contrast, respectively. No difference was observed for pattern-onset in the same kernel at the same latency for these two types of contrast.

Two other previous studies were able to isolate the chromatic response elicited by patterned pseudo-random stimulation (Baseler and Sutter, 1997; Gerth et al., 2003). Baseler and Sutter (1997) observed positive-dominated K2.1 waveforms for pattern-reversal equiluminant red-green dartboards, while Gerth et al. (2003) described negative-dominated K1 for pattern-onset and negative-dominated K2.1 waveforms for pattern-reversal equiluminant red-green triangle arrays, respectively.

The polarity of pseudo-random luminance VECPs depends on stimulus configuration. K2.1 with positive polarity at 100 ms was observed with central single hexagon stimulus of high luminance contrast (Klistorner et al., 1997) as well as with high luminance contrast pattern-reversal sinusoidal gratings (Araújo et al., 2013). However, when triangle patterns were used, luminance pattern-onset K1 had negative polarity, similarly to chromatic pattern-onset K1 (Gerth et al., 2003). We found no difference between color blinds and normal trichromats in the amplitude of luminance pattern-onset and pattern-reversal VECP kernels.

Conventional chromatic VECP recorded from congenital red-green color-blinds were used to evaluate if the response would be dependent of color-opponent mechanisms (Kinney and McKay, 1974; Regan and Spekreijse, 1974; Crognale et al., 1993; Suttle and Harding, 1999; Gomes et al., 2006, 2008). All these studies have found that VECP had small amplitude or was entirely absent for color contrast modulated along color confusion axes. In this work, we observed that the amplitudes at 100 ms of VECP kernels obtained from color blinds by using compound stimuli were much smaller than from normal trichromats. Some color blind subjects exhibited small positivity at 100 ms evoked by compound pattern-reversal stimuli, probably reflecting the activation of luminance mechanisms.

Visual evoked cortical potential kernels elicited by pattern-reversal and pattern-onset stimuli might not represent the same state of adaptation of the visual system (Sutter, 2000). We used base periods of 13.3 and 26.6 ms for pattern-reversal and pattern-onset stimuli, respectively. The pattern-reversal K2.2 is equivalent to the pattern-onset K2.1, once both of them represent the interaction between two stimulus impulses separated by 26.6 ms. The statistical differences of the VECP amplitude between trichromats and color-blinds for pattern-reversal K2.1, and pattern-reversal K2.2 give support to the suggestion that these kernels are generated by similar chromatic mechanisms.

## CONCLUSION

In conclusion, we suggest that compound pattern-reversal K2.1 is the best stimulus configuration to differentiate between luminance and chromatic mechanisms in VECP studies.

## Conflict of Interest Statement

The authors declare that the research was conducted in the absence of any commercial or financial relationships that could be construed as a potential conflict of interest.
